# Preparing Generation Z of Health Professions for Artificial Intelligence Revolution Through Hacking Education: An Interventional Study

**DOI:** 10.1002/hsr2.71169

**Published:** 2025-08-18

**Authors:** Hossein Rezazadeh, Ali Madadi Mahani, Mahla Salajegheh

**Affiliations:** ^1^ Endocrinology and Metabolism Research Center Kerman University of Medical Sciences Kerman Iran; ^2^ Student Committee of Medical Education Development, Education Development Center Kerman University of Medical Sciences Kerman Iran; ^3^ Department of Medical Education, Medical Education Development Center Kerman University of Medical Sciences Kerman Iran

**Keywords:** artificial Intelligence, generation Z, hack in education, medical education

## Abstract

**Background and Aims:**

The integration of artificial intelligence into healthcare is rapidly expanding, yet formal AI education for health profession students remains limited. Generation Z students, as digital natives, require innovative instructional methods tailored to their learning preferences. This study aimed to investigate the effectiveness of an innovative course based on hacking education for Z‐generation health profession students about artificial intelligence.

**Methods:**

This was a single‐group pre‐ and post‐test interventional study conducted with 81 health profession students. Educational needs were identified through an expert panel. A 100‐h flipped classroom course incorporating group discussions, communities of practice, peer teaching, and gamification was delivered. Pre‐ and post‐course questionnaires assessed students' familiarity with key AI concepts.

**Results:**

Post‐course assessments showed a significant improvement in students' familiarity with AI‐related domains, including programming languages (*p* < 0.001), machine learning (*p* < 0.001), AI applications in healthcare (*p* < 0.001), data science (*p* < 0.001), image processing (*p* < 0.001), deep learning (*p* < 0.001), neural networks (*p* < 0.001), and statistics (*p* < 0.001).

**Conclusion:**

The hacking education‐based AI course effectively enhanced students' AI competencies. Given the increasing role of AI in healthcare, integrating structured AI training into medical curricula is essential to prepare future healthcare professionals for AI‐driven clinical environments.

## 1 Introduction

In recent decades, novel technological developments have led to the emergence and spread of new techniques in diagnosing and treating diseases in medical science. This is because some conventional and traditional methods are likely no longer adequate for meeting the needs of physicians and patients in the process of healthcare practice [1]. One of the novel areas of technology in the healthcare profession is the emergence and application of artificial intelligence (AI). AI in the context of health professions education involves the study and application of computer algorithms and models that enable machines to analyze complex medical data, recognize patterns, make predictions, and assist in clinical decision‐making. This includes the use of programming languages like Python to develop AI applications, machine learning techniques for data analysis, and deep learning algorithms for tasks such as image processing and neural network modeling [2]. AI applications in the healthcare profession focus primarily on diagnostic support, treatment planning, patient monitoring, and operational enhancements within medical education [3]. AI has transformed medical technologies and can aid in processing medical data, providing healthcare professionals with important insights, and improving health outcomes and patient experiences [4].

Studies conducted on the use of AI in the healthcare profession indicate the potential capacity of this technology to improve different aspects of healthcare practice directly relevant to medical education including disease diagnosis, treatment selection, health management, and patient care [5], but there remains a gap in medical education regarding how future healthcare professionals are trained to use these technologies effectively.

Given the global expansion of AI, it is expected that AI will be one of the core elements of medical education in the coming years [6] to equip students with the knowledge and skills necessary to utilize AI technologies in their future professions [7]. Therefore, teaching the principles of AI and its application in the healthcare profession becomes very important for medical students as future healthcare professionals who will benefit from AI in their daily clinical practice [8]. However, current medical curricula often lack formal AI training, leaving students underprepared for AI‐integrated healthcare environments.

Chen et al. (2022) revealed that while most physicians and medical students are aware of the increasing use of clinical AI, they lack practical experience and relevant knowledge. Further training is needed to facilitate the change and adoption of these new technologies [9]. Most medical schools do not offer structured AI courses, leaving students without systematic exposure to AI tools and applications. Moreover, AI education in healthcare is often theoretical rather than practical, providing minimal hands‐on experience with AI‐driven decision‐making, data analysis, or machine learning techniques [10]. Liu et al. (2022) declared that formal curricula and current resources for AI are limited at most US medical schools, and there is a definite knowledge gap in AI education in current US medical training [11]. In the curricula of health profession education in Iran's medical education system, there is no program for teaching AI and its application in healthcare professions to students. This is while the study results indicate the effectiveness of training provided in this regard to students. Sit et al. (2020) stated that medical students trained in AI feel more confident about working with AI in the future than those who did not receive this training [12]. Park et al. (2021) showed that formal AI education for medical students helps combat misinformation and prevent dissuading them from learning about and applying AI in their future professional practice [13]. Buabbas et al. (2023) revealed that most students believed that learning about AI would benefit their careers, and all medical students need to receive education on AI [14].

On the other hand, the majority of students currently enrolled in the education system are Generation Z students who have certain characteristics, such as a strong interest in digital communications and media, a preference for interactive, technology‐based learning, and a preference for collaborative and interactive teaching methods [15, 16]. Therefore, education planners and faculty must take into account the unique characteristics of these students when planning and providing education on AI in the healthcare profession [17, 18]. Focusing specifically on their comfort with technology and active learning strategies is essential for designing effective AI courses. In addition, one of the concepts that has recently become a trend in education and encompasses new teaching methods that could address the educational needs of Generation Z students is the concept of hacking in education [19]. Hacking education refers to the use of creative, technology‐driven strategies to innovate traditional learning environments. It involves rethinking conventional teaching methods by incorporating elements such as gamification, project‐based learning, and hackathons to foster active student engagement. At first glance, the meaning that comes to mind about this concept seems negative and related to the computer world. However, in recent years, hacking has been transforming into part of educational reforms as well [20, 21]. In education, the concept of “hack” has become popular as educators seek innovative ways to engage students and improve learning outcomes. As Wizel (2019) described “hack in education” involves applying hacker mentality and techniques, such as using technology creatively and challenging traditional structures, to promote innovation within the educational system [20]. These hacking techniques encompass various strategies like gamification, hackathons, creating new tools and resources for education, use of multimedia presentations, online forums, and educational apps for project‐based learning [19]. Butt et al. (2020) demonstrated the effectiveness of hack in education in promoting cross‐disciplinary learning in medical education [22]. By challenging rigid teaching models and incorporating AI‐related problem‐solving activities, hacking education presents an effective approach to bridging the gap between AI knowledge and practical application. In this sense, hacking the traditional education system through utilizing new teaching methods, strengthening collaboration and critical thinking, and employing creativity and innovation in education leads to increased student participation and more effective teaching and learning [22, 23]. Therefore, this study aimed to investigate the effectiveness of an innovative course based on hacking education for the Z‐generation of health profession students about AI.

## 2 Methods

### 2.1 Study Design and Setting

This single‐group pre‐ and post‐test study was conducted at Kerman University of Medical Sciences from February 2023 to May 2023.

### 2.2 Study Participants and Sampling

The data were collected from 81 health profession students at Kerman University of Medical Sciences who entered the study by the census method. Students were invited via messenger apps to participate in this voluntary, extracurricular course about AI. The inclusion criteria consisted of an interest in AI in the healthcare profession. The exclusion criteria included failure to complete relevant prerequisite courses, failure to complete assignments on time, and dropping out of the course.

### 2.3 Program Design

The program was designed by adopting the ASSURE model as a procedural guide for planning and delivering instructions integrating technology into the teaching process [24]. According to this model, the characteristics of learners, aim and objectives, educational strategies, teaching methods utilizing methods, media, and materials, participation of learners, and student performance evaluation were addressed.

To identify the general characteristics of the learners, we distributed a questionnaire including items regarding age, sex, field of study, phase of study (undergraduate or postgraduate), year of study, and previous courses or experience related to AI among the volunteers.

To inform the development of an AI educational program catered to medical students, a review of pertinent literature was conducted alongside the engagement of a four‐member expert panel using the nominal group technique. Participants included two medical informatics professors, one AI professor, and one AI PhD candidate, recruited via email and in‐person invitation. The panel's objective entailed identifying essential AI training needs for the medical student audience. Utilizing the nominal group technique, experts first privately documented insights responding to the central question, “What are the educational needs of health professional students about AI?”. Afterward, a round‐robin format enabled members to voice their ideas to the group. Next, facilitated discussion of the proposed needs allowed participants to clarify and vote on priorities. Ultimately, the panel's output and literature review findings were presented to a separate assembly of four medical education and AI experts to delineate the resultant program's overarching purpose and goals.

Based on the identified educational needs, learning objectives were specified and formulated. These objectives included acquiring theoretical knowledge and practical skills in the concepts, applications, and modeling of AI. Some examples of different kinds of learning objectives include:

- Define key terms related to AI and its applications in health professions.
- List different programming languages commonly used in AI, focusing on Python.
- Interpret the impact of AI on healthcare outcomes and patient care.
- Illustrate the process of data preprocessing and feature extraction in AI applications using healthcare data sets.
- Apply machine learning techniques to analyze healthcare data sets and derive insights.
- Design a prototype AI model for a specific healthcare use case, such as medical image classification or predictive diagnostics.
- Analyze the ethical implications of AI decision‐making in clinical settings.

Then, based on the defined objectives, instructional content comprising basic and applied concepts of AI was prepared. This content was extracted from authoritative and up‐to‐date resources in the field of AI. Additionally, student‐centered and interactive teaching strategies were selected for delivering the course. In the next stage, diverse instructional materials and resources, such as texts, instructional videos, software, and application data sets, were prepared for presenting the content.

### 2.4 Program Implementation

A longitudinal, integrated program performed in a community of practice setting was chosen based on situated learning theory [25]. After the announcement of the program, 95 students registered voluntarily, and 81 of them participated in the program.

To clarify the integration of the “hacking education” concept into the program design, we explicitly linked hacking principles to our methods. The hacking education approach, which emphasizes creative problem‐solving, student autonomy, and innovation, directly informed the design of our teaching strategies. These strategies included:

- The flipped classroom: 8 2‐h synchronized sessions were held after presenting the instructional content to the students, allowing them to resolve issues, discuss, and elaborate on the topics—fostering problem‐solving and self‐directed learning, core principles of hacking education.
- Face‐to‐face instructional sessions: 20 5‐h sessions were conducted through lecturing, group discussion, and hands‐on methods. In line with hacking education, participants collaborated in groups of 20, with each group assigned a mentor to encourage innovation and guide students through AI challenges.
- Communities of practice (COPs): Monthly group projects encouraged collaboration and real‐world problem‐solving, consistent with hacking education's focus on teamwork and experimentation. Students worked in groups of 4 on AI projects, participating in competitions such as Kaggle.
- Peer teaching: Reflecting the hacking ethos of knowledge sharing, near‐peer tutors led “Small Sessions,” empowering students to teach and learn from one another.
- Gamification: Inspired by hacking strategies, a gamified system awarded points and medals for completing assignments and advancing through tasks, enhancing engagement and motivation.

To further clarify the program structure, we have added Table 1 which summarizing the modes of teaching, their duration, and objectives.

**Table 1:** Summarize of the modes of teaching, their duration, and objectives.

Teaching modality	Duration	Objective
Flipped classroom	8 sessions, 2 h each	Foster self‐directed learning, problem‐solving, and discussion of AI concepts.
Face‐to‐face sessions	20 sessions, 5 h each	Provide hands‐on AI training, group discussions, and mentorship to support practical learning.
Communities of practice (COPs)	Monthly group projects	Encourage collaboration and real‐world AI problem‐solving by working in small teams and participating in competitions (e.g., Kaggle).
Peer teaching	Weekly “Small Sessions”	Promote collaborative learning by allowing top‐performing students to tutor their peers and strengthen collective AI understanding.
Gamification	Ongoing throughout the program	Increase motivation and engagement by awarding points and medals for completing assignments, advancing through levels, and achieving higher ranks.

### 2.5 Program Evaluation

A pretest–posttest design enabled the measurement of participant learning and the intervention's efficacy. Participants completed a tailored questionnaire pre‐ and post‐course covering core educational content via 10 5‐point Likert scales (strongly agree to strongly disagree, with 5 being the highest) with multiple‐choice questions and five subjective open‐ended prompts gauging satisfaction and attitudes towards the program. The face validity of the questionnaire were confirmed through a pilot test with 10 medical students to ensure clarity and content relevance. The content validity was verified by a panel of four AI and medical education experts. The reliability of the questionnaire was confirmed by calculating Cronbach's alpha coefficient which obtained 0.95.

Questionnaires were electronically distributed at the program conclusion, and a follow‐up reminder was sent 2 weeks later via email and social media to maximize the response rate.

Statistical analysis was conducted using SPSS. Paired *t*‐tests were used to compare pre‐ and post‐test scores. The qualitative evaluation analyzed through thematic analysis. Additionally, at the end of the course, a test of course theory discussion and an individual practical project were held for each student.

### 2.6 Ethical Consideration

The research study underwent ethical review and approval from the National Agency for Strategic Research in Medical Education's Ethical Review Board, as documented by identifier IR. NASRME. REC.1402.023. Participation remained entirely voluntary, without any participant incentives. Following ethics committee‐approved procedures, researchers obtained both verbal and written informed consent from participants. The study was non‐compensated and confidential, with data usage strictly limited to the research aims outlined in the ethics review application. All participant information was protected according to confidentiality assurances.

## 3 Results

### 3.1 Participants' Demographics

A total of 81 health profession students participated in the AI educational program after registration. The participants' demographics are presented in Table 2.

**Table 2:** Participants' demographics.

Variable		Number	Percentage
Gender	Female	56	69.14
	Man	25	30.86
Field of study	Medicine	24	29.63
	Medical informatics	11	13.5
	Health information technology	10	12.35
	Radiology	5	6.17
	Environmental health engineering	5	6.17
	Pharmacology	3	3.70
	Medical mycology	3	3.70
	Health	2	2.47
	Clinical Biochemistry	2	2.47
	Nursing	2	2.47
	Nutrition science	2	2.47
	Librarianship and medical information	2	2.47
	Occupational health and safety engineering	2	2.47
	General hygiene	1	1.23
	Dentistry	1	1.23
	Health policy	1	1.23
	Medical physics	1	1.23
	Science and food industry engineering	1	1.23
	Computer engineering	1	1.23
	Hematology	1	1.23
	Anesthesiology	1	1.23
Age		Average	Standard deviation
		24.78	7.08

### 3.2 Questionnaire Response Rate

In the evaluation phase, the response rate to the post‐course questionnaire was 84%. We have also accounted for missing data by employing pairwise deletion for incomplete responses, ensuring that all valid data points were included in the statistical analyzes. Of the 95 students who initially registered, 14 did not complete the course. We have mentioned the dropout rate explicitly and have adjusted our analyzes using pairwise deletion for missing data.

### 3.3 Pre‐ and Post‐Test Results

**Figure 1:**
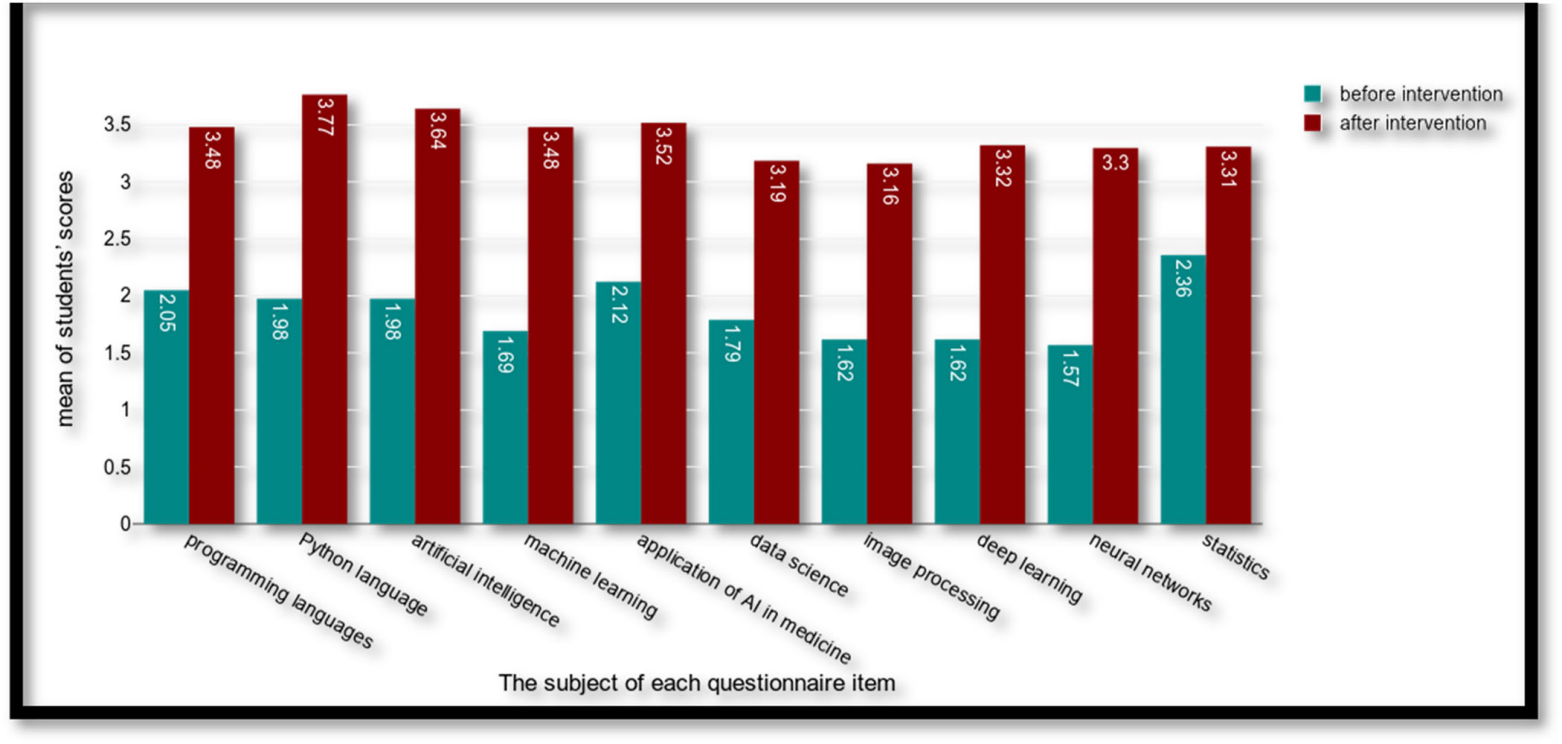
Comparing the level of students' knowledge in artificial intelligence before and after intervention.

Most participants (95.8%) stated that their motivation to take part was an interest in AI or the relation of this topic for their future careers. A comparison of students' pretest and posttest scores regarding AI in the health profession demonstrated the effectiveness of the instruction provided. As evident from the Table 3 results, there was a significant difference between the students' scores about familiarity with programming languages, Python language, AI, machine learning, application of AI in medicine, data science, image processing, deep learning, neural networks in AI, and statistics before and after the educational intervention (*p* value less than 0.001). Considering the means obtained, their understanding and familiarity improved following the training intervention (Figure 1).

**Table 3:** Comparison of students' scores with artificial intelligence before and after intervention.

Question	Pretest		Posttest		*p* value
	Mean	SD	Mean	SD	
To what extent were you familiar with programming languages before participating in this course?	2.05	1.03	3.48	0.91	< 0.001
To what extent were you familiar with Python language before participating in this course?	1.98	1.04	3.77	0.87	< 0.001
To what extent were you familiar with artificial intelligence before participating in this course?	1.98	0.92	3.64	0.87	< 0.001
To what extent were you familiar with machine learning before participating in this course?	1.69	1.08	3.48	0.93	< 0.001
To what extent were you familiar with the application of AI in medicine before participating in this course?	2.12	0.98	3.52	0.93	< 0.001
To what extent were you familiar with data science before participating in this course?	1.79	1.06	3.19	0.96	< 0.001
To what extent were you familiar with image processing before participating in this course?	1.62	0.94	3.16	1.11	< 0.001
To what extent were you familiar with deep learning before participating in this course?	1.62	0.88	3.32	1.01	< 0.001
To what extent were you familiar with neural networks in AI before participating in this course?	1.57	0.85	3.30	1.12	< 0.001
To what extent were you familiar with statistics before participating in this course?	2.36	1.15	3.31	0.98	< 0.001

### 3.4 Practical Projects

Participants also completed practical projects during the course. These projects included:

Designing AI models for disease detection: Students developed basic machine learning algorithms for medical image classification.
Data analysis for clinical predictions: Groups analyzed healthcare data sets to predict patient outcomes using AI techniques.
Kaggle competitions: Several student groups participated in AI challenges on Kaggle, applying their newly acquired skills in real‐world scenarios.

### 3.5 Qualitative Analysis of Open‐Ended Prompts

- To assess participants' subjective experiences, open‐ended prompts were analyzed using thematic analysis. Responses were coded into themes, including:
- Increased confidence: Many participants reported feeling more confident in their ability to engage with AI concepts.
- Desire for further learning: Several students expressed interest in more advanced AI training.
- Challenges faced: Some participants noted difficulties with certain technical aspects, such as Python programming.

## 4 Discussion

The purpose of this study was to investigate the effectiveness of an innovative course based on hacking education for the Z generation of health profession students about AI. The results indicate that the implementation of an innovative course based on hacking education is a viable option for teaching AI to Z‐generation students. Therefore, our research seeks evidence to answer the need revealed by many researchers that AI competencies of health profession education students should be improved [26], as there is abundant student interest in this topic [27].

Using the ASSURE model as a practical guide for development and delivering training provided high‐quality evidence for program design. Cai et al. (2023) applied the ASSURE model to enable smart teaching by using AI and digital resources. They revealed that the ASSURE model provides a full and clear procedural guide for digitalized instructional design [28]. Lei (2023) discusses the new development concept of educational technology based on the ASSURE model and demonstrates its effectiveness in serving the needs of cultivating new capacities [29]. While it has shown effectiveness in various educational contexts, its success may be influenced by cultural and infrastructural factors. Malik et al (2023) highlights that the effectiveness of Islamic religious education is enhanced through the ASSURE model, which integrates media and technology, such as PowerPoint and smartphones, to actively engage students and tailor learning to their characteristics and objectives. While supportive factors include increased student confidence, easier comprehension, and improved teacher assessment, challenges such as tardiness and low student motivation hinder the learning process [30]. Future studies should explore how the model performs in resource‐limited settings or among populations with varying levels of technological literacy.

Gen Z as authentic digital natives think and process information fundamentally differently than prior generations and exhibit high levels of collaboration, independence, and practicality [15, 31]. Some theories claim that the best teaching and learning methods for these learners should contain aspects such as material rewards, the possibility of increasing their power and prestige, encouraging work, enriched settings, and recognition as individuals [32]. One of the most effective actions is the investment in hacking education to offer new ways of teaching and learning for students of Gen Z. The concept of hacking education addresses innovative educational methods, reinforcing collaboration and critical thinking, using creativity and innovation in education, which leads to more participation of learners and more effective learning [22]. Therefore, our intervention benefited flipped classrooms, group discussions, COP, near‐peer tutors, and gamification.

In our study, the application of CoP integrated with gamification was a key feature. There are three main perspectives on the role of CoPs. Some see them as platforms for enabling learning and expertise exchange. Others view them primarily as drivers of innovation. A third lens focuses on how CoPs consolidate control and protect domain interests [33, 34]. We used the first two lenses in our research to make sense of the ways CoPs are conceptualized as AI. Our findings revealed that our participants were better engaged in sharing their knowledge and experiences to perform the projects. This strategy connected them with their peers to build sustainable communities. Frehywot and colleague (2023) believed that partnerships in health profession training need to be developed in ways that enable the formation of an equitable and sustainable CoP to address the use of AI for global health workforce training [35]. Wang and colleague (2020) used CoPs as a knowledge management strategy to support education in embracing AI. They claimed that the collaborative learning process in CoPs has made individuals make sense of AI core concepts [36]. Despite these positive outcomes, future research should further investigate how CoPs impact not only knowledge sharing but also the practical application of AI concepts in clinical settings, ensuring that learners can translate theoretical understanding into real‐world practice.

One of the strengths of this study was the incorporation of gamification with the content of the program. Kim and colleague (2019) proposed a curriculum that helped students learn AI in board game form. They determined that gamification enables learners to develop their ability to understand objects from various perspectives and enhance their thinking skills [37]. Yordanova (2020) reviewed the gamification literature and argued that gamification may be in use for AI development [38]. Soboleva et al. (2021) applied gamification to train some elements of AI, including algorithmization and programming. They clarified that gamification in learning AI can improve the quality of students' educational results [39]. However, our study primarily focused on students' familiarity with AI concepts rather than their actual competency. Future studies should incorporate objective measures of AI skills to evaluate whether gamification translates into concrete improvements in AI proficiency.

One of the other advantages of our intervention was the application of the near‐peer tutor method. We argue that this educational method was useful for training students in the field of AI in healthcare professions. This method, which was designed based on Bandura's (1977, 1997) theorization, is defined as a relationship between a mentor and mentee, who are proximal in age but somewhat distant in expertise [40]. Our results are consistent with the findings of Sun and colleague (2022), who developed a near‐peer mentoring model for recruiting youth into computer science. Their findings provided evidence for the effectiveness of the model in improving self‐efficacy in computer science [41]. Nonetheless, in our study, the small sample sizes within certain subgroups, such as hematology and dentistry, limit the generalizability of these findings. Larger and more diverse samples in future studies will be necessary to validate the effectiveness of the near‐peer tutor method across different health professions.

Cultural constraints can significantly impact the adoption and implementation of innovative educational methods. Some of our limitations regarding cultural factors in the use of student‐centered approaches were lack of compliance with standards appropriate to the innovative learning approach, such as classroom equipment. There are other similar experiences in our country. Hemmati and colleague (2022) explored teachers' understanding of the obstacles to implementing student‐centered approaches and revealed the unawareness of educational managers, teachers, and students, inappropriate instructional content, lack of necessary expertise among the teachers, lack of attention to planning appropriate curriculum and material development, and funding problems [42]. Moradi and colleague (2020) investigated the status of learner‐centered instruction in Iranian language schools and found that resource constraints and teachers' tendency toward applying traditional practices as the two overriding barriers restricting proper learner‐centered methods. Poor motivation on the part of teachers was identified as the most prominent barrier [43]. Frambach et al (2014) investigate how in student‐centered education, students' cultural backgrounds are expressed in discussions in East Asia, Western Europe, and the Middle East. They revealed that student‐centered education is feasible in different cultural contexts, but across these contexts, processes and outcomes are likely to differ [44].

Although most of the educational methods that we applied are frequently used in different settings, they are almost innovative for our setting which the majority of educational methods are yet teacher‐centered. There are many contexts whose educational conditions are similar to ours, that's our intervention and its results can be useful for these societies. Considering the lack of any formal educational program for teaching AI and its application in the healthcare profession in Iranian curricula, this study is the first effort to train health profession education students regarding AI.

## 5 Limitations

One of the limitations was that most participants stated that they took part in the course because of their intrinsic interest in AI. This self‐selection bias may have led to an overestimation of the course's effectiveness. Another limitation is the small sample size and lack of long‐term follow‐up of students. Implementing longitudinal studies would provide deeper insights into the sustained impact of AI education. Also, our results are interpreted without a control group. Future studies should incorporate control groups or comparison groups to better isolate the impact of the intervention. We acknowledge that the use of messenger apps for recruitment may have inadvertently excluded students less engaged with digital communication tools, which is recognized as a limitation of this study. Although census sampling was used, we recognize the potential for selection bias, as participation may have been influenced by self‐motivation or a pre‐existing interest in AI. Given the small sample sizes within certain subgroups (e.g., hematology, dentistry), we acknowledge the challenge of drawing statistically meaningful conclusions about the program's impact within these fields. Future iterations of the program could benefit from conducting more detailed subgroup analyzes by field of study, gender, or other demographic variables to tailor AI education strategies more effectively. One of the other limitations is the rapid change and advancement in AI, and some of the findings may not be generalizable or citable in future years. However, the fundamental principles and novel educational methods utilized in this study can serve as effective approaches for teaching AI to Generation Z students. Also, while self‐reported familiarity provides insight into students' perceived learning, future studies should incorporate performance‐based assessments (e.g., coding assignments, AI model development, or clinical case simulations) to evaluate the actual application of AI knowledge in real‐world scenarios.

## 6 Conclusion

AI skills training for health profession students is becoming increasingly relevant. Therefore, we performed an innovative course based on hacking education, including flipped classrooms, group discussions, CoPs, near‐peer tutors, and gamification, for the Generation Z health profession students about AI. The findings indicated that this course significantly improved participants' AI competencies. After this course, students became better acquainted with programming languages, machine learning algorithms, and practical applications of AI in the healthcare field.

## Author Contributions

**Hossein Rezazadeh:** conceptualization, investigation, methodology, writing – original draft, writing – review and editing. **Ali Madadi Mahani:** conceptualization, investigation, data curation, writing – review and editing, writing – original draft. **Mahla Salajegheh:** conceptualization, writing – review and editing, writing – original draft, supervision, formal analysis.

## Ethics Statement

The research study underwent ethical review and approval from the National Agency for Strategic Research in Medical Education's Ethical Review Board, as documented by identifier IR. NASRME. REC.1402.023. Participation remained entirely voluntary, without any participant incentives. Following ethics committee‐approved procedures, researchers obtained both verbal and written informed consent from participants. The study was non‐compensated and confidential, with data usage strictly limited to the research aims outlined in the ethics review application. All participant information was protected according to confidentiality assurances.

## Consent

The authors have nothing to report.

## Conflicts of Interest

The authors declare no conflicts of interest.

## Transparency Statement

The lead author Mahla Salajegheh affirms that this manuscript is an honest, accurate, and transparent account of the study being reported; that no important aspects of the study have been omitted; and that any discrepancies from the study as planned (and, if relevant, registered) have been explained.

## Data Availability

The data that support the findings of this study are available on request from the corresponding author. The data are not publicly available due to privacy or ethical restrictions.

## References

[1] P. Liu , L. Lu , J. Zhang , T. Huo , S. Liu , and Z. Ye , “Application of Artificial Intelligence in Medicine: An Overview,” Current Medical Science 41 (2021): 1105–1115. 34874486 10.1007/s11596-021-2474-3 PMC8648557

[2] R. Charow , T. Jeyakumar , S. Younus , et al., “Artificial Intelligence Education Programs for Health Care Professionals: Scoping Review,” JMIR Medical Education 7, no. 4 (2021): e31043. 34898458 10.2196/31043 PMC8713099

[3] A. Sharma , S. Rani , and D. Gupta , “Artificial Intelligence‐Based Classification of Chest X‐Ray Images Into COVID‐19 and Other Infectious Diseases,” International Journal of Biomedical Imaging 2020 (2020): 1–10. 10.1155/2020/8889023 PMC7539085 33061946

[4] H. Rezazadeh , A. M. Mahani , and M. Salajegheh , “Insights Into the Future: Assessing Medical Students' Artificial Intelligence Readiness‐A Cross‐Sectional Study at Kerman University of Medical Sciences (2022),” Health Science Reports 8, no. 5 (2025): e70870. 40432697 10.1002/hsr2.70870 PMC12106343

[5] S. A. Alowais , S. S. Alghamdi , N. Alsuhebany , et al., “Revolutionizing Healthcare: The Role of Artificial Intelligence in Clinical Practice,” BMC medical education 23, no. 1 (2023): 689. 37740191 10.1186/s12909-023-04698-z PMC10517477

[6] P.‐S. Goh and J. Sandars , “A Vision of the Use of Technology in Medical Education After the COVID‐19 Pandemic,” MedEdPublish 9, no. 49 (2020): 49. 38058893 10.15694/mep.2020.000049.1 PMC10697445

[7] J. Krive , M. Isola , L. Chang , T. Patel , M. Anderson , and R. Sreedhar , “Grounded in Reality: Artificial Intelligence in Medical Education,” JAMIA Open 6 (2023): 6. 10.1093/jamiaopen/ooad037 PMC10234762 37273962

[8] A. H. Sapci and H. A. Sapci , “Artificial Intelligence Education and Tools for Medical and Health Informatics Students: Systematic Review,” JMIR Medical Education 6, no. 1 (2020): e19285. 32602844 10.2196/19285 PMC7367541

[9] M. Chen , B. Zhang , Z. Cai , S. Seery , M. J. Gonzalez , N. M. Ali , et al., “Acceptance of Clinical Artificial Intelligence Among Physicians and Medical Students: A Systematic Review With Cross‐Sectional Survey,” Frontiers in Medicine 9 (2022): 990604. 36117979 10.3389/fmed.2022.990604 PMC9472134

[10] R. Tolentino , A. Baradaran , G. Gore , P. Pluye , and S. Abbasgholizadeh‐Rahimi , “Curriculum Frameworks and Educational Programs in Ai for Medical Students, Residents, and Practicing Physicians: Scoping Review,” JMIR Medical Education 10, no. 1 (2024): e54793. 39023999 10.2196/54793 PMC11294785

[11] D. S. Liu , J. Sawyer , A. Luna , et al., “Perceptions of US Medical Students on Artificial Intelligence in Medicine: Mixed Methods Survey Study. JMIR,” Medical Education 8, no. 4 (2022): e38325. 10.2196/38325 PMC9636531 36269641

[12] C. Sit , R. Srinivasan , A. Amlani , et al., “Attitudes and Perceptions of UK Medical Students Towards Artificial Intelligence and Radiology: A Multicentre Survey,” Insights into imaging 11 (2020): 14. 32025951 10.1186/s13244-019-0830-7 PMC7002761

[13] C. J. Park , P. H. Yi , and E. L. Siegel , “Medical Student Perspectives on the Impact of Artificial Intelligence on the Practice of Medicine,” Current Problems in Diagnostic Radiology 50, no. 5 (2021): 614–619. 32680632 10.1067/j.cpradiol.2020.06.011

[14] A. J. Buabbas , B. Miskin , A. A. Alnaqi , et al., “Investigating Students' Perceptions Towards Artificial Intelligence in Medical Education,” Healthcare. 11 (2023): 1298. 37174840 10.3390/healthcare11091298 PMC10178742

[15] M. Hernandez‐de‐Menendez , C. A. Escobar Díaz , and R. Morales‐Menendez , “Educational Experiences With Generation Z,” International Journal on Interactive Design and Manufacturing (IJIDeM) 14 (2020): 847–859.

[16] A. Szymkowiak , B. Melović , M. Dabić , K. Jeganathan , and G. S. Kundi , “Information Technology and Gen Z: The Role of Teachers, the Internet, and Technology in the Education of Young People,” Technology in Society 65 (2021): 101565.

[17] G. A. Talmon , “Generation Z: What's Next?,” Medical Science Educator 29, no. Suppl 1 (2019): 9–11. 10.1007/s40670-019-00796-0 PMC8368917 34457613

[18] M. Salajegheh , S. N. Hekmat , and M. Macky , “Challenges and Solutions for the Promotion of Medical Sciences Faculty Members in Iran: A Systematic Review,” BMC Medical Education 22, no. 1 (2022): 406. 35619090 10.1186/s12909-022-03451-2 PMC9134687

[19] F. Barpi , D. Dalmazzo , A. De Blasio , and F. Vinci , “Hacking Higher Education: Rethinking the EduHack Course,” Education Sciences. 11, no. 2 (2021): 40.

[20] M. Wizel Teachers as Hackers: Implications for 21st Century Teacher Education. 7th Teaching and Education Conference; 2019; Lesley University.

[21] M. Wizel Teachers as Hackers: Implications for 21st Century Teacher Education: Lesley University; 2017.

[22] W. A. Butt , Q. Shahood , W. H. Farooqi , K. Ghias , S. Sabzwari , and A. Mian , “Healthcare Hackathons: Fostering Medical Education Through Innovation in a Developing Country: A Case Study From Pakistan,” BMJ Innovations. 7, no. 1 (2020): 1–6.

[23] J. K. Wang , S. K. Roy , M. Barry , R. T. Chang , and A. S. Bhatt , “Institutionalizing Healthcare Hackathons to Promote Diversity in Collaboration in Medicine,” BMC medical education 18, no. 1 (2018): 269. 30458759 10.1186/s12909-018-1385-x PMC6245929

[24] J. R. Bajracharya , “Instructional Design and Models: ASSURE and Kemp,” Journal of Education and Research 9, no. 2 (2019): 1–8.

[25] I. Pyrko , V. Dörfler , and C. Eden , “Communities of Practice in Landscapes of Practice,” Management Learning 50, no. 4 (2019): 482–499.

[26] D. Wiljer , M. Salhia , E. Dolatabadi , et al., “Accelerating the Appropriate Adoption of Artificial Intelligence in Health Care: Protocol for a Multistepped Approach,” JMIR Research Protocols 10, no. 10 (2021): e30940. 34612839 10.2196/30940 PMC8529463

[27] E. A. Wood , B. L. Ange , and D. D. Miller , “Are We Ready to Integrate Artificial Intelligence Literacy Into Medical School Curriculum: Students and Faculty Survey,” Journal of Medical Education and Curricular Development 8 (2021): 23821205211024078. 34250242 10.1177/23821205211024078 PMC8239949

[28] J.‐Y. Cai , R.‐L. Ma , and J. Xu Application of ASSURE Model in International Chinese Language Smart Education. Proceedings of the 2023 6th International Conference on Big Data and Education; 2023.

[29] G. Lei , “Influence of Assure Model in Enhancing Educational Technology,” Interactive Learning Environments 32, no. 7 (2023): 3297–3313.

[30] M. I. Malik , M. S. Nugraha , and Tarsono , “Analysis Implementation of the ASSURE Model in Enhancing the Effectiveness of Islamic Religious Education Learning,” Andragogi: Jurnal Ilmiah Pendidikan Agama Islam 5, no. 2 (2023): 121–134.

[31] K. Lesinskis , I. Mavlutova , A. Spilbergs , and J. Hermanis , “Digital Transformation in Entrepreneurship Education: The Use of a Digital Tool KABADA and Entrepreneurial Intention of Generation Z,” Sustainability. 15, no. 13 (2023): 10135.

[32] J. Kalpakian and A. Marzouk , “Generation Z: Implications for Universities,” ELite Journal: International Journal of Education, Language, and Literature 3, no. 1 (2023): 35–45.

[33] D. Nicolini , I. Pyrko , O. Omidvar , and A. Spanellis , “Understanding Communities of Practice: Taking Stock and Moving Forward,” Academy of Management Annals 16, no. 2 (2022): 680–718.

[34] A. Abedini , B. Abedin , and D. Zowghi , “Adult Learning in Online Communities of Practice: A Systematic Review,” British Journal of Educational Technology 52, no. 4 (2021): 1663–1694.

[35] S. Frehywot and Y. Vovides , “An Equitable and Sustainable Community of Practice Framework to Address the Use of Artificial Intelligence for Global Health Workforce Training,” Human Resources for Health 21, no. 1 (2023): 45. 37312214 10.1186/s12960-023-00833-5 PMC10262492

[36] T. Wang and C. K. E. Cheng Thinking Aloud and Progressing Together: Cultivating Communities of Practice for Supporting Hong Kong K‐12 Schools in Embracing Artificial Intelligence. The International Conference on Education and Artificial Intelligence 2020 (ICEAI) 2020.

[37] J. Kim and N. Park , “Development of a Board Game‐Based Gamification Learning Model for Training on the Principles of Artificial Intelligence Learning in Elementary Courses,” Journal of The Korean Association of Information Education 23, no. 3 (2019): 229–235.

[38] Z. Yordanova Gamification as a Tool for Supporting Artificial Intelligence Development–State of Art. International Conference on Applied Technologies; 2019: Springer.

[39] E. V. Soboleva , T. N. Suvorova , A. V. Grinshkun , and M. I. Bocharov , “Applying Gamification in Learning the Basics of Algorithmization and Programming to Improve the Quality of Students' Educational Results,” European Journal of Contemporary Education 10, no. 4 (2021): 987–1002.

[40] S. Irvine , B. Williams , and L. McKenna , “Near‐Peer Teaching in Undergraduate Nurse Education: An Integrative Review,” Nurse Education Today 70 (2018): 60–68. 30145536 10.1016/j.nedt.2018.08.009

[41] C. Sun and J. Clarke‐Midura , “Testing the Efficacy of a Near‐Peer Mentoring Model for Recruiting Youth Into Computer Science,” Mentoring and Tutoring: Partnership in Learning 30, no. 2 (2022): 184–201.

[42] M. R. Hemmati and F. Aziz Malayeri , “Iranian EFL Teachers’ Perceptions of Obstacles to Implementing Student‐Centered Learning: A Mixed‐Methods Study,” International Journal of Foreign Language Teaching and Research 10, no. 40 (2022): 133–152.

[43] M. R. Moradi and P. Alavinia , “Learner‐Centered Education In the Iranian EFL Context: A Glance Through the Impediments,” Teaching English as a Second Language Quarterly (Formerly Journal of Teaching Language Skills) 38, no. 4 (2020): 95–121.

[44] J. M. Frambach , E. W. Driessen , P. Beh , and C. P. M. Van der Vleuten , “Quiet or Questioning? Students' Discussion Behaviors in Student‐Centered Education Across Cultures,” Studies in Higher Education 39, no. 6 (2014): 1001–1021.

